# Wind Turbine Blade Surface Roughening and Plastic Emission Due to Leading Edge Erosion: Multiple Impact Modeling Framework

**DOI:** 10.3390/ma19050963

**Published:** 2026-03-02

**Authors:** Antonios Tempelis, Leon Mishnaevsky

**Affiliations:** Department of Wind and Energy Systems, Technical University of Denmark, 4000 Roskilde, Denmark

**Keywords:** rain erosion, wear, wind turbine blades, microplastics, high-speed impact

## Abstract

This paper presents a multiple water droplet impact finite element model that can be used to simulate high strain rate water droplet erosion processes for various target materials. The model is able to provide predictions for mass loss and the evolution of erosion depth as a function of the number of impacts. This is achieved through a continuum damage mechanics approach coupled with element deletion for the target material. Validation of the model is performed by comparison with water droplet erosion data for PMMA. We apply the model to estimate the emissions of microplastics from wind turbines due to blade erosion. For adverse weather and operational conditions, our worst-case estimate was to the order of 340 g per blade per year. The developed framework is also used to model the effect of flaws in the blade coating on erosion progression. The effect of internal defects (voids) in the coatings on the erosion depth evolution was studied numerically. The presence of internal voids led to earlier coating breakthrough and exposure of the substrate material. The model can be used to study the effects of various types of flaws during both the incubation and mass loss stages of erosion.

## 1. Introduction

Recently, surface erosion of wind turbine blades has been identified as a source of microplastic emissions [1,2]. Surface erosion is caused by high-speed (80–120 m/s) impacts of rain droplets, hail and sand, which damage the polymer coatings that are used to protect the blades [3,4] and lead to the detachment of plastic particles, which end up in the environment. For offshore wind turbines, the plastic particles are highly likely to fall into the sea. The term microplastic is used due to the eroded particles often having a size of less than 5 mm [5,6]. These particles can have detrimental effects on the health of living organisms, and they can be stored within human bodies as they travel up the food chain or travel through the air. Incredibly small particles are able to directly penetrate cells and organs [5,6,7].

Recent studies have provided estimations for the average emissions of microplastics from wind turbine blades per year. Caboni et al. [1] used an empirical model coupled with experimental rain erosion testing data and provided estimations to the order of 240 g per turbine per year for the Dutch North Sea. Mishnaevsky Jr. et al. [2] used repair data and empirical modeling and made estimates of 80–1000 g per turbine per year for offshore turbines. In both studies, the reported amounts were orders of magnitude lower than those from other pollution sources, such as car tires. In an effort to understand the erosion process and determine important mechanical properties that can lead to improved coating performance, mechanical models can be quite useful tools. The Springer model [8] combines a fatigue damage approach with the load from each water droplet impact, which is determined through the water hammer equation and coating elastic properties, to provide estimations for coating lifetimes. The model can be extended by an empirical approach to provide mass loss predictions for coatings as a function of the number of impacts. Castorrini et al. [9] used the mass loss equation of the Springer model to estimate the depth of erosion at various locations along a blade. Other studies have used simulations to determine the load in the coating [10,11,12,13,14,15] and then transfer the stresses to a fatigue damage accumulation equation to perform lifetime estimations [10,16,17]. Walayat et al. [18] used the peridynamics approach to study solid particle impacts and the mass loss for a few (five) particle impacts. Ashrafizadeh et al. [19] used a finite element model with element deletion to study solid particle impacts and determine the effect of temperature on the erosion rate of polyurethane coatings, again for a small number of impacts.

However, there is a general lack of simulation approaches that model the mass loss stage of erosion due to multiple water droplet impacts. The rain erosion process takes place during hundreds or thousands of impacts, and this is impossible to perform within a single simulation. During the mass loss stage, the impact surface is constantly changing due to material failure, and this can alter the pressure profiles that are applied by each droplet impact. Also, a model that dives deeper into the micromechanics of droplet impacts and damage formation in the coating than an empirical model can provide a better understanding of the mass loss stage. It can also allow for evaluating the effects of various flaws within the coating on its anti-erosion performance. Furthermore, such a model can be used for determining the emitted mass of microplastics from wind turbines and for establishing pollution mitigation strategies. Therefore, the aims of this paper are the following:

1. Present a finite element modeling framework that explicitly simulates thousands of impacts one by one and accounts for the constantly evolving surface geometry during the mass loss stage;
2. Simulate the erosion depth evolution on the protective coating as a function of the number of impacts through an element deletion approach;
3. Use the developed framework to make predictions for microplastic emissions from blades due to rain erosion;
4. Study the effect of coating flaws such as voids and air bubbles on the mass loss stage of the erosion process.

As stated, the mass loss stage will be modeled through element deletion, which is governed by a damage variable that is defined for each element of the coating layer. A result transfer technique will be used to simulate a large number of impacts, where each impact is treated in a separate simulation and the surface geometry for the coating is updated for each simulation. This technique has been previously used in wear and abrasion simulations for sliding contact conditions [20,21,22,23,24,25]. The Archard equation [26] is frequently used for such cases to update the surface heights according to the applied pressure. In the case of water droplet erosion, the Archard equation may not be directly applicable, and we use a continuum damage mechanics approach, which also accounts for fatigue loading of the coating material. This approach deletes elements once a critical damage value has been reached. Similar approaches for wear simulations have been used by Leonard et al. [24] and Morales-Espejel and Brizmer [25]. Raimondo and Cini [27] used an equivalent plastic strain-based approach to simulate erosion by a few thousand impacts while running only a limited number of simulations. Even though a continuum damage mechanics approach is still an empirical relationship, it relates the external loading conditions to the internal damage developed within the material through the internal stresses and strains that are developed. In general, the parameters of the damage equation for this case can be considered material properties for the coating. Thus, they are not influenced by the size or the speed of the impacting droplets. This offers the advantage that any kind of loading can be applied, and the stresses and strains will determine how fast and at which location the damage develops. Therefore, this approach offers versatile predictions and can be used to study how various droplet sizes, angles and impact speeds affect the mass loss of the coating. Also, various flaw geometries that influence the developed stresses and strains locally can be introduced into the coating to study how they affect the performance.

This paper is organized as follows. In Section 2, the droplet impact finite element model and the damage accumulation equation are introduced. This section also describes the result transfer method between impact simulations and how a flaw in the form of an air void is introduced into the model. Modern coating materials for blades are often viscoelastic, and the material model used to account for their load rate sensitivity is also described in this section. The model is validated in Section 3 with water droplet erosion data for PMMA. In Section 4, the model is applied for erosion of wind turbine blade coatings, and the predicted erosion geometry and erosion depth evolution curves are presented and compared between the non-flawed and flawed coating cases. The erosion depth evolution curves are then used to make predictions for the emitted microplastic volume per turbine and are shown to be similar to values previously reported in the literature. Section 5 and Section 6 present a discussion and the conclusions of this study, respectively.

## 2. Methods

### 2.1. Basic Droplet Impact Model

**Figure 1:**
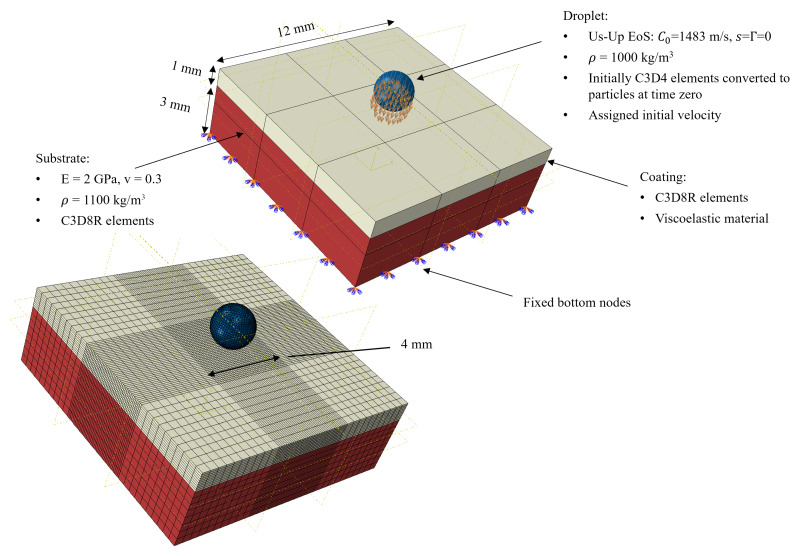
Droplet impact finite element model geometry, mesh and some modeling details.

The finite element (FE) simulation domain consists of a small representative volume of the leading edge protection system and the rain droplet. The model is presented in Figure 1. The impact of the droplet is simulated with an explicit dynamic solver in ABAQUS, using the smoothed-particle hydrodynamics (SPH) approach to resolve the large droplet deformations [10]. The FE mesh and types of elements are also presented in Figure 1.

**Figure 2:**
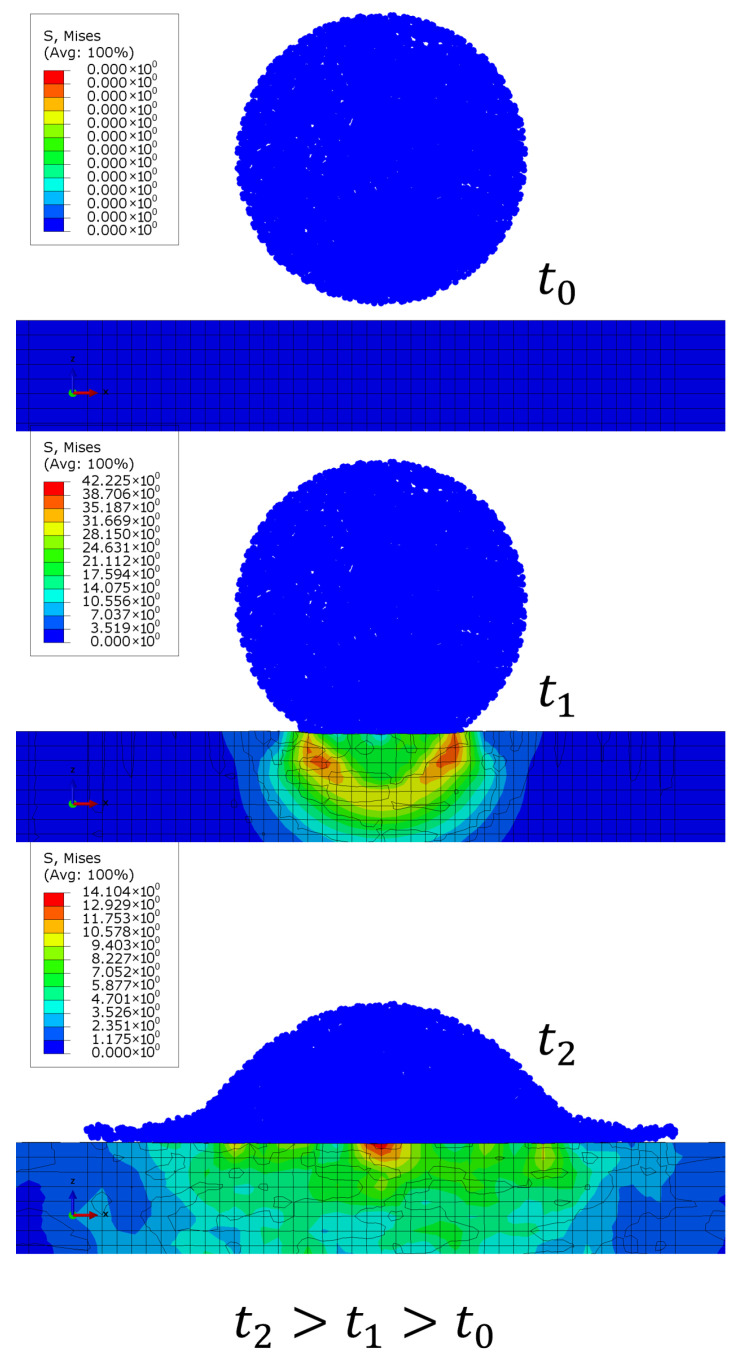
An example of the evolution of the von Mises stress field in the coating during the impact using SPH particles for the droplet. Stress is expressed in MPa.

The droplet is initially meshed with continuum elements which are converted to particles at the beginning of the simulation (Figure 2). A linear-viscoelastic material model is used for the top coating layer, as will be described in Section 2.4, and a linear elastic polymeric material is used to model the substrate. The coating density is taken to be equal to 1100 kg/m^3^, considering a polyurethane-based material.

The water droplet is modeled with a material model based on the linear Us-Up equation of state [28], which can account for the formation of shock waves traveling inside the droplet and also for the effects of compressibility during high-speed water droplet impact. The constants of the equation of state in ABAQUS are $C_{0}$ = 1483 m/s, Γ = 0 and *s* = 0. No viscosity value is assigned as the flow is expected to be near inviscid due to inertial effects dominating over surface tension effects [29]. A contact with a “hard” normal overclosure and a frictionless tangential interaction in ABAQUS is defined between the droplet and the coating.

### 2.2. Modeling Failure and Element Deletion for the Coating Layer

During the impact, a dynamically evolving stress field is developed in the coating, as shown in Figure 2. To simulate failure of the coating layer and volume loss, elements are deleted based on a damage variable. This damage variable describes the growth of micro-damages within each element, and when a critical damage value is reached, material is detached from the coating. This process evolves over multiple droplet impacts, and we use a nonlinear damage accumulation equation for each element, which accounts for stress triaxiality effects as well [30,31]. The damage evolution equation is

(1) $$\frac{dD}{dN}={\left(\frac{\sigma_{max,vm}}{m(1-D)}\right)}^{a+b}R_{v}^{b/2}$$

where *D* is the damage variable, dD/dN is the damage increment for the current impact, *m*, *a* and *b* are material parameters that control damage accumulation, $\sigma_{max,vm}$ is the maximum von Mises stress recorded in the element for the current impact and

(2) $$R_{v}=\frac{2}{3}\left(1+v\right)+3\left(1-2v\right){\left(\frac{\sigma_{max,H}}{\sigma_{max,vm}}\right)}^{2}$$

where *v* is the Poisson’s ratio of the material and $\sigma_{max,H}$ is the maximum hydrostatic stress recorded in the element during the impact. In the denominator of the term inside the parentheses of the damage evolution equation, *D* refers to the damage value at the end of the previous impact.

The damage variable was updated at the last time increment of the analysis based on the maximum von Mises and stress triaxiality values during the impact event. The simulation was performed for a sufficient amount of time so that the stress field fully developed over time and the maximum stress values were reached. By deleting elements only at the last time increment, dynamic stress redistribution effects, which might trigger excessive and unwanted element deletion, were avoided.

As elements are deleted after successive impacts, sharp interior corners are formed that might lead to undesirable stress singularities and unbounded stress increases. This effect may lead to unrealistically fast damage accumulation and premature element deletion. To account for this, a limit stress value was introduced into the damage calculations, which was considered to be the yield stress of the material at the relative loading rates and was equal to 120 MPa. Thus, if the maximum von Mises stress exceeds this value, then the yield stress of 120 MPa is used in the damage equation. A stress threshold value of 10 MPa was also considered, below which no damage was accumulated. The damage equation was incorporated inro the FE model through a VUSDFLD subroutine.

### 2.3. Multiple Impact Modeling Framework

Erosion of the protective coating was observed after hundreds or thousands of impacts. The process involves an initially undamaged surface during the incubation period and a dynamically evolving surface as erosion progresses further. The dynamically evolving surface will influence the stress field and also the damage accumulation within each element. This needed to be considered in the simulations.

**Figure 3:**
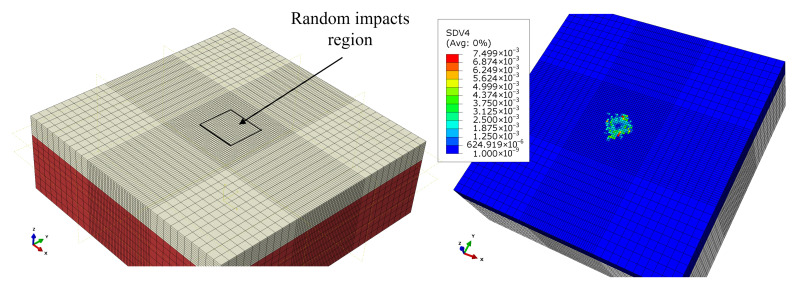
Schematic of the region where random droplet positions are generated and the fatigue damage field variable (SDV4) contour at the surface after the first impact.

First, to simulate a random rain load, the central droplet axis for each impact was positioned randomly within a rectangular area of dimensions 1 mm by 1 mm. Damage was accumulated as the number of impacts increased, and eventually element deletion occurred. The size of the impacted area and an example damage accumulation contour after the first droplet impact are presented in Figure 3. The modeling framework treated each impact as a separate event, and the results were transferred between simulations through a combination of the user subroutines VUSDFLD and VEXTERNALDB.

**Figure 4:**
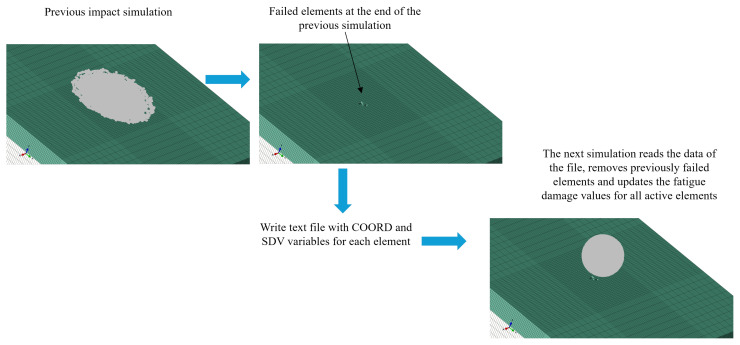
Example of how damage and failed elements are transferred between impact simulations using the subroutines VEXTRENALDB and VUSDFLD.

After the end of the first impact simulation, the state variables (SDVs) that store the accumulated damage, element failure flag variable and coordinates of the corresponding material point (COORD variable of one material point per element as reduced integration elements were used) were stored in a text file. The next simulation read the text file through the VEXTERNALDB subroutine and mapped the accumulated damage or deleted failed elements in the new simulation through the VUSDFLD subroutine, based on the COORD values. This process is illustrated in Figure 4, and it was repeated until the target number of impacts was achieved. This process allows for simulating the evolution of erosion depth as a function of the number of impacts by recording the maximum depth at which an element has been deleted. All impacts occurred at a normal angle to the surface. It was assumed that there was no interaction between two successive droplet impacts and the stress field diminished before the next impact occurred. However, the interaction between impacts and impact rate effects could be included by importing the deformed coating configuration and the material state (stresses and strains) at the end of the previous simulation.

**Figure 5:**
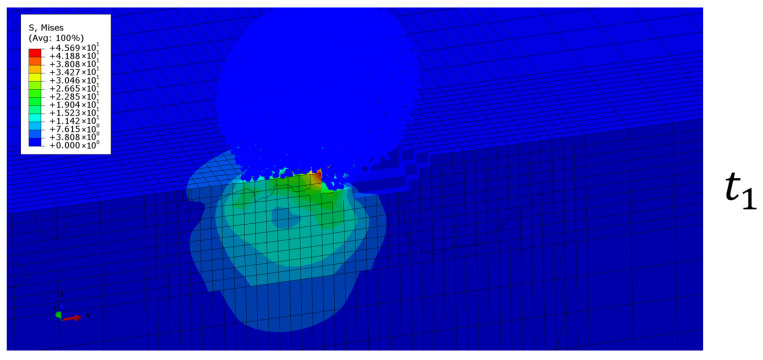

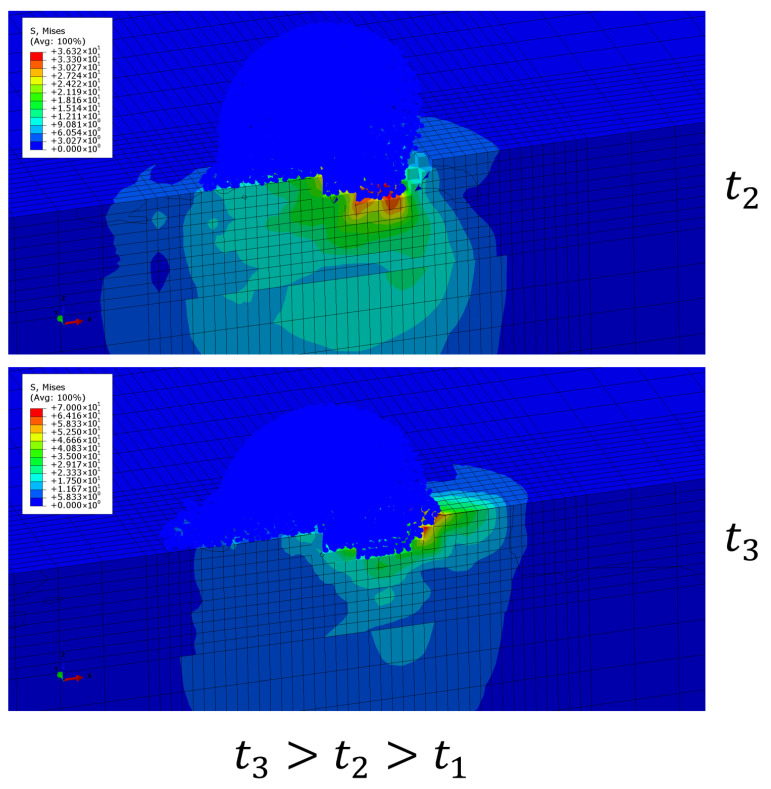
Interaction between droplet jetting and eroded geometry at different times during the impact event. Von Mises stress expressed in MPa.

This simulation framework can also capture the interaction between the droplet and the damaged (or roughened) coating surface and how that changes the stress field (Figure 5). This is important as jetting effects of the droplet can increase the stresses and lead to faster damage accumulation [32]. To capture this effect, the general contact algorithm in ABAQUS must also include the interior faces of the coating elements so that the contact inclusions are updated each time an element fails.

The element length of the fine mesh area in the simulations was 0.1 mm. The same global size was used for the initial mesh of the droplet. The modeling framework is expected to simulate thousands of impacts, and the computational demand is high. The mesh size of 0.1 mm was found to provide an acceptable tradeoff between computational time and convergence for the stresses. For these types of simulations, a mesh size equivalent to 300 elements per droplet diameter (or 0.01 mm for a 3 mm droplet diameter) was required to reach stress convergence when using a coupled Eulerian-Lagrangian approach [32]. Therefore, since we used a coarser mesh, the parameters of the damage accumulation equation that will be used in Section 4.1 were calibrated for a mesh size of 0.1 mm and expected to be different for a finer mesh size.

### 2.4. Viscoelastic Blade Coating Material Model

The top coating is often viscoelastic, and its mechanical response is loading rate- and temperature-dependent. In this work, we only consider the dependency on the loading rate by using a linear viscoelastic material model based on data from dynamic mechanical analysis (DMA) tests. The data from DMA comes in the form of master curves which describe the ability of the coating to store elastic energy (storage modulus) and dissipate energy due to viscoelastic effects (loss modulus), over a range of loading rates, at a reference temperature. The data was obtained by loading the material with small strains at different loading frequencies and temperatures under shear, and a master curve was constructed for a reference temperature of 0 °C using the time-temperature superposition principle with appropriate shift factors. The test data was obtained for a polyurethane-based coating.

**Figure 6:**
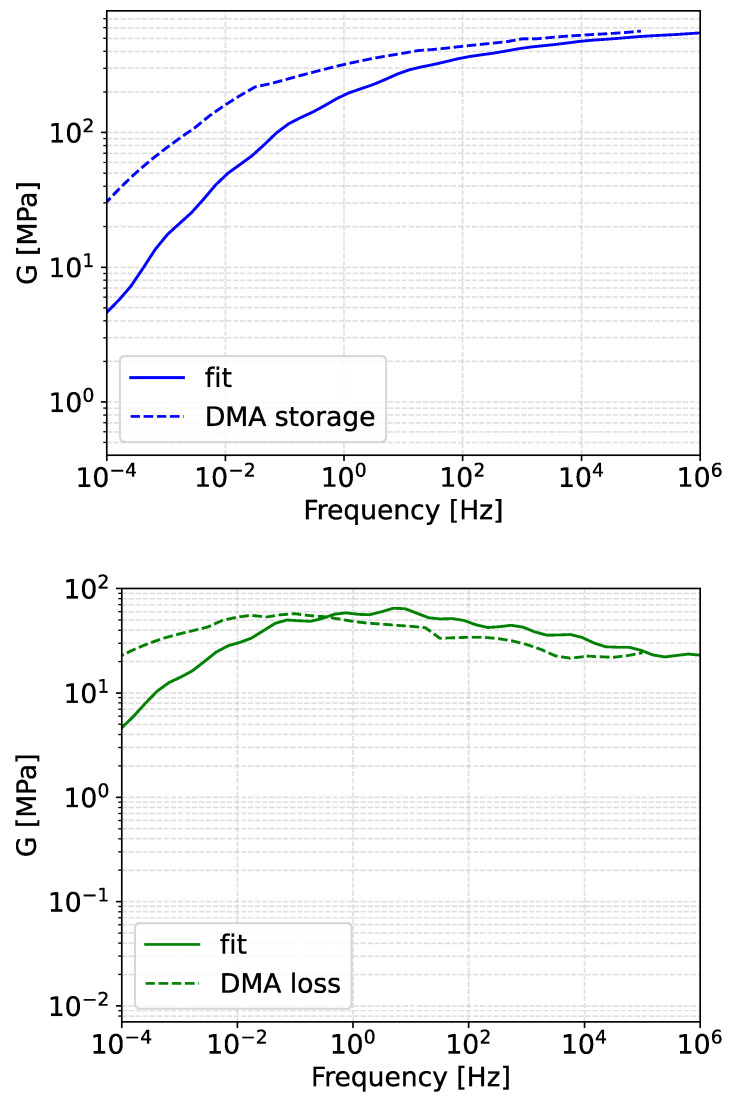
Storage and loss shear moduli master curves and the corresponding fit of a linear-viscoelastic material model for a reference temperature of 0 °C.

The DMA data is presented in Figure 6. Also, in Figure 6, the fit of the DMA master curves by a linear elastic material coupled with Prony series in ABAQUS to account for viscoelasticity is presented. The Prony series provide the time dependency of the elastic modulus such that

(3) $$E\left(t\right)=E_{0}\left[1-\sum_{i=1}^{N_{p}}g_{i}\left(1-e^{-t/\tau_{i}}\right)\right]$$

where $E_{0}$ is the instantaneous (glassy state) modulus, $N_{p}$ is the number of Prony series terms, $g_{i}$ and $\tau_{i}$ are the parameters of each term and *t* is time.

The fitted material model is described by an instantaneous elastic modulus of $E_{0}$ = 1680 MPa and a Poisson’s ratio of *v* = 0.4. The Prony series parameters were obtained with the help of MCalibration software (version 7.2.2), and they are presented in Table 1.

**Figure 7:**
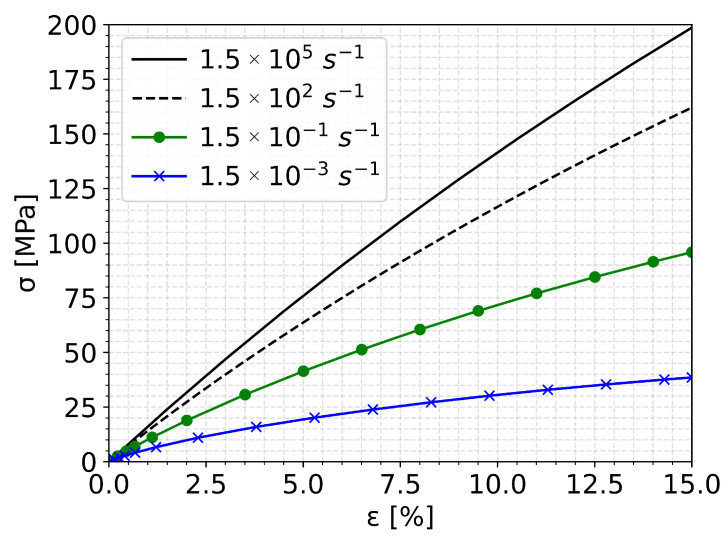
Loading rate-dependent stress-strain curves of the linear-viscoelastic coating model obtained with single-element FE simulations.

During rain droplet impacts, the strain rates in the coating can exceed $10^{5}$ s^−1^. The linear-viscoelastic material model allows for capturing the effect of loading rates, under the assumption of small strains, so that the material behaves in a linear viscoelastic manner. In Figure 7, for example, the stress–strain curves of the considered material model up to a strain of 15% for various loading rates are presented. These curves were obtained by single-element FE models in ABAQUS, using the Viscous time step analysis type.

The material model captured the rate dependency of the stress–strain curves, although validation was not possible due to a lack of high strain rate data. Capturing this dependency is important, as different loading rates are experienced at different points in the coating during droplet impact, and the stress values used in the damage equation were significantly influenced by the loading rate.

### 2.5. Void in the Coating Layer

**Figure 8:**
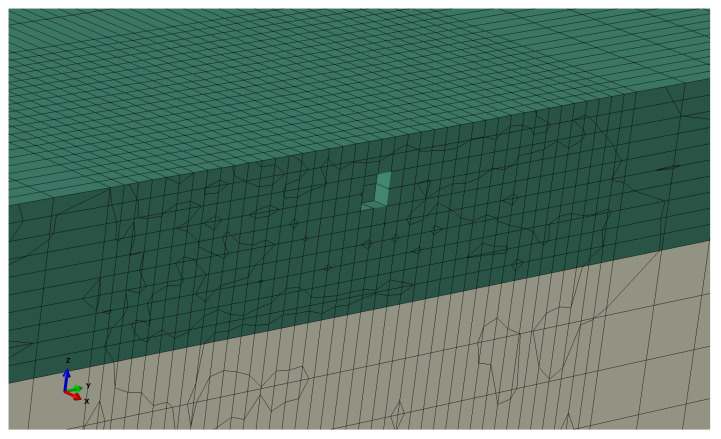
Cross-section of the coating layer that shows the void geometry used in the multiple impact framework.

Voids in the form of air bubbles can be formed inside the coating layer during curing or manufacturing. Such voids are believed to reduce the performance of coatings during rain erosion testing. The effect of air bubbles during the incubation period has been studied numerically with FE simulations by Pandey et al. [30] and experimentally by Jensen et al. [33] and Fæster et al. [34]. Using the modeling framework of this paper, we want to investigate how the presence of a void can influence the post-incubation behavior of the coating. For this purpose, we approximated a void geometry by removing a set of elements from the coating layer, as shown in Figure 8.

The void diameter was equal to 0.2 mm, placed at a distance of 0.2 mm from the coating’s top surface and along the central axis of the simulation domain. The damage equation and its parameters account for the presence of small voids in the microstructure of the material, which are uniformly distributed in space. However, bigger flaws such as the void considered here must either be modeled explicitly, as shown in Figure 8, or indirectly by reducing the critical damage value for certain elements. The latter approach can be more useful in cases where the size of a void or flaw is slightly smaller than the element size used in the simulation.

## 3. Validation

For validation of the developed multi-impact numerical model, experimental data for water droplet erosion of cast PMMA from the study by Wang et al. [35] was used. Specifically, an experiment with a constant droplet impact speed of 325 m/s and a droplet diameter of 1.5 mm was modeled. All droplet impacts in the experiment could be considered to occur at the same position, and therefore, a model where all droplet impacts occurred at the center of the target surface was constructed. The thickness of the PMMA target was 6 mm. For the elastic properties of the cast PMMA, the values *E* = 3.23 GPa and *v* = 0.4 were used, considering an isotropic material response [35]. Model output and experimental data in the form of erosion depth versus the number of impacts (*N*) and eroded surface profiles will be compared.

For the validation simulations, the following damage accumulation per impact equation was used, where the effects of triaxiality were not considered, according to Equation (4):

(4) $$\frac{dD}{dN}=C{\left(\frac{S_{max}}{A(1-D)}\right)}^{B}$$

where, again, $S_{max}$ is the maximum von Mises stress recorded at each element during the whole impact event and *A*, *B* and *C* are parameters. The advantage of using this equation is that its parameters can be fitted in a way that a given number of impacts to failure can be obtained for a certain stress level. Since the impact position was the same for all impacts, and no surface geometry changes occurred during the erosion incubation period, the largest $S_{max}$ value over all elements in the model could be used to fit the parameters so that the incubation period was the same as the experimental one. The maximum $S_{max}$ value was 252 MPa in the finite element model for an impact speed of 325 m/s, considering an element size of 0.1 mm. From the experimental curve of depth versus number of impacts for 325 m/s from [35], a value of about 7000 impacts corresponds to the incubation period. Then, Equation (4) with a constant value of $S_{max}$ = 252 MPa was used within an optimization algorithm, with appropriate initial values for parameters *A*, *B* and *C*. The optimization process fits the values of *A*, *B* and *C* so the damage value *D* becomes equal to one after 7000 impacts. The optimized values were *A* = 432 MPa, *B* = 7.387 and *C* = 9.05 × 10^−4^.

Since the impact position did not change from impact to impact, cycle jumping could be applied to reduce the number of required simulations. Cycle jumping was applied with a constant cycle jump value $N_{jump}$ between impact numbers i−1 and *i* according to Equation (5):

(5) $$D^{i}=D^{i-1}+\frac{dD}{dN}N_{jump}$$

**Figure 9:**
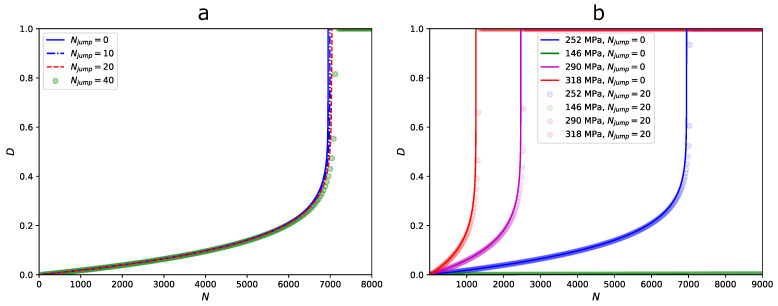
Effect of cycle jumping on fatigue damage accumulation: (**a**) maximum stress of 252 MPa and (**b**) multiple stress levels.

In Figure 9a, the damage accumulation for a constant value of $S_{max}$ = 252 MPa is plotted without considering cycle jumping and with three values of $N_{jump}$. We observe that the difference between the damage accumulation curves increased as the value of $N_{jump}$ increased, with reference to the curve for $N_{jump}$ = 0. The difference became significant when the value of $N_{jump}$> 20. In Figure 9b, the damage evolution curves for $N_{jump}$ = 0 and $N_{jump}$ = 20 are plotted for various values of $S_{max}$. The cycle jumping approach was observed to be valid for larger and smaller levels of $S_{max}$.

**Figure 10:**
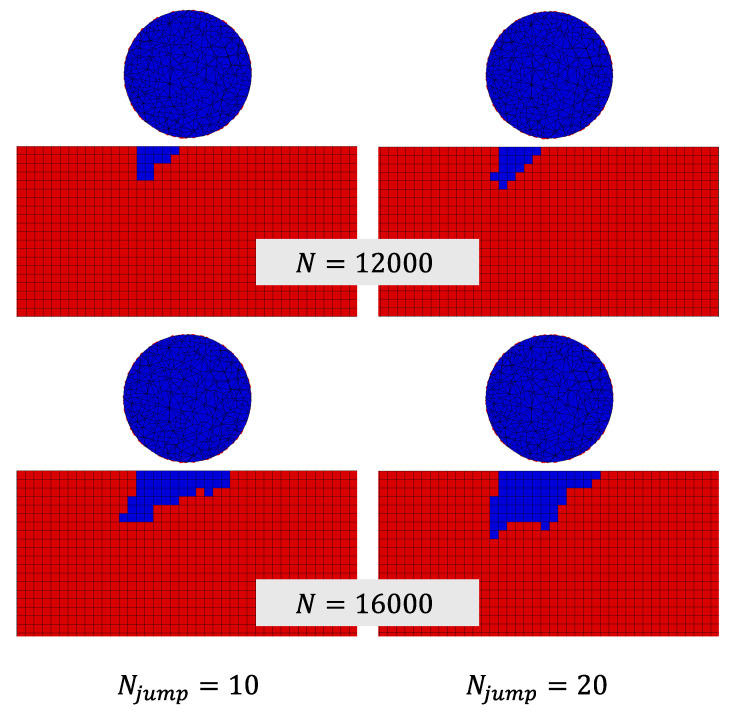
Simulated eroded cross-section after 12 × 10^3^ and 16 × 10^3^ impacts with two cycle jump values. Eroded elements are marked in blue, and the remainder is coated in red.

Since the considered material model was linear elastic, we introduced a stress limit to account for the possibility of stress singularities. This limit was set at 400 MPa, which we considered to be the strength of PMMA at high strain rates. If the computed $S_{max}$ value at each element is greater than this value, then $S_{max}$ = 400 MPa in Equation (4). The resulting erosion profiles at the middle cross-section of the target domain after running the multi-impact finite element model for $N_{jump}$ = 10 and $N_{jump}$ = 20 are presented in Figure 10 after 12 × 10^3^ and 16 × 10^3^ impacts. The eroded elements are marked in blue, and the droplet diameter is also plotted for reference.

**Figure 11:**
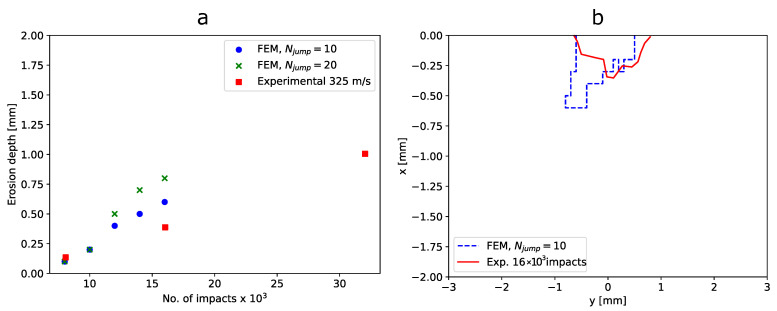
(**a**) Comparison between experimental and simulated erosion depth evolution [35] for two cycle jump values. (**b**) Comparison between experimental [35] and simulated erosion profile after 16 × 10^3^ impacts.

While the profiles were similar to each other, they were somewhat different, which is attributed to the redistribution of stresses once material erosion occurs. A smaller value of $N_{jump}$ might be required for higher accuracy. The simulated maximum erosion depth as a function of the number of impacts for $N_{jump}$ = 10 and $N_{jump}$ = 20 was compared to the experimental curve from [35] in Figure 11a. The simulated erosion profile at the middle of the target domain was compared to a characteristic erosion profile from the experiments of [35] after 16 × 10^3^ impacts in Figure 11b. We observe that the multi-impact finite element model can provide accurate results when provided accurate input parameters.

## 4. Results

### 4.1. Calibration of the Damage Equation Parameters

**Figure 12:**
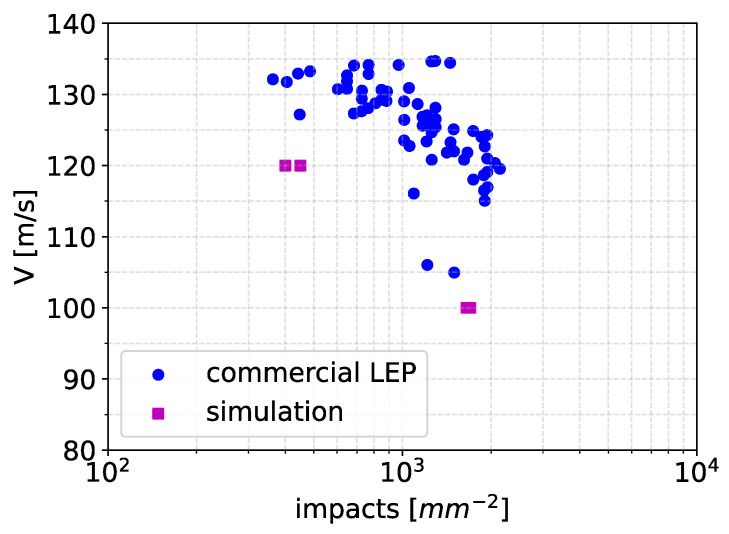
Impact speed versus number of impacts to failure (*V*-*N* graph) for a commercial coating system [36] and a comparison with the performance of the material considered in the simulations.

The model is now applied to viscoelastic blade protection coatings. Cycle-jumping was not used. The damage equation parameters were calibrated based on the rain erosion testing data of a commercial leading edge protection (LEP) coating [36]. The test data represents the impact speed versus the number of impacts to failure (*V*-*N*) datapoints obtained by a whirling arm rain erosion tester [37]. The datapoints are presented in Figure 12, where the horizontal axis is expressed in terms of impacts per mm^2^. The end of incubation for the coating, i.e., when the first signs of damage were observed during testing, was used to determine the datapoints in the graph and in Table 2.

With a trial-and-error approach, parameters *m*, *a* and *b* of the damage accumulation equation were varied so that the simulation predictions were within the range of the experimental data. The values of *m* = 200 MPa, *a* = 1.3 and *b* = 5.7 were found to provide the results presented in Figure 12. In fact, these values provide predictions of a performance considerably worse than that of the commercial coating system. Therefore, the predictions for the erosion depth and volume loss of the coating were expected to be conservative. Two impact speeds of 120 m/s and 100 m/s were considered in the simulation, and three runs were performed for each speed to account for randomness in the impact positions within the simulations. There existed a small scatter in the predictions of the simulation. The droplet diameter for both cases was 2.4 mm, equal to the average droplet size used in the rain erosion test. The end of incubation in the simulations was calculated as the impact number when the first element failed.

### 4.2. Erosion Depth Evolution

**Figure 13:**
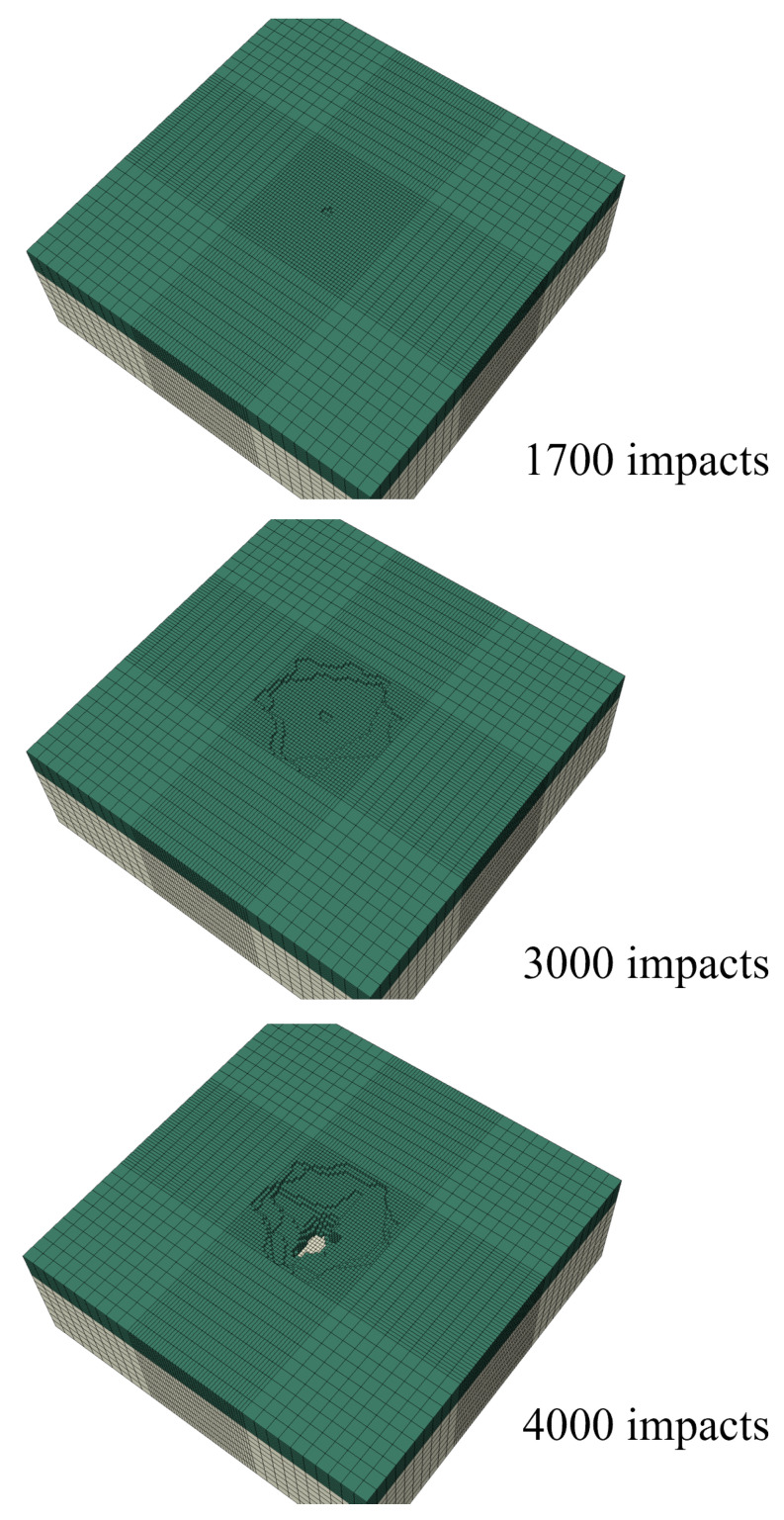
Evolution of surface erosion in the simulation for an impact speed of 100 m/s and 2.4 mm droplet diameters.

By using the developed framework and modeling past the incubation period, it is possible to obtain the evolution of the maximum erosion depth as a function of the number of impacts. The maximum erosion depth was determined by the failed element with the maximum through-thickness coordinate value. The damage equation parameters of Section 4.1 were used, with an impact speed of 100 m/s and a 2.4 mm droplet diameter for all impacts. The evolution of the eroded surface for the considered parameters is presented in Figure 13.

**Figure 14:**
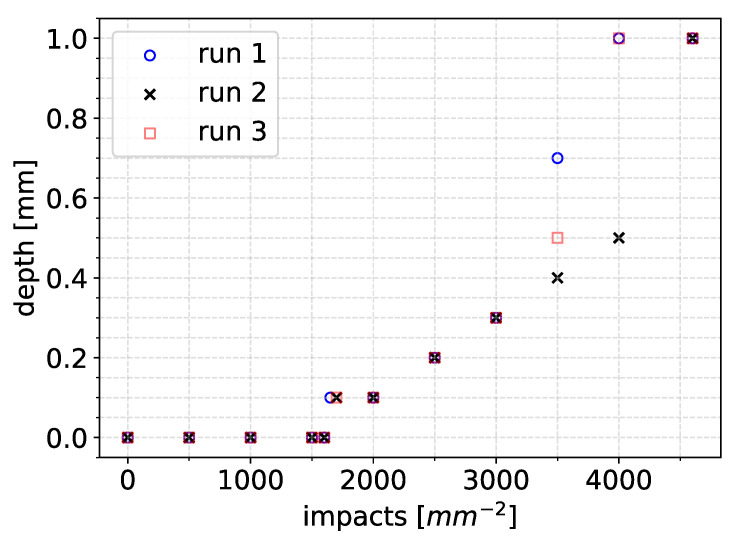
Evolution of the maximum erosion depth in the three runs of the simulation for an impact speed of 100 m/s and 2.4 mm droplet diameters.

The simulation was stopped soon after full breakthrough of the coating layer and exposure of the substrate layer was achieved. Since the impact positions were random, some scatter in the results was expected. To check this scatter, three runs were performed. The resulting maximum erosion depth versus time graphs are presented in Figure 14.

The end of incubation was recorded between 1500 and 1700 impacts per mm^2^ for all three runs. The maximum depth evolved almost linearly until about 3500 impacts per mm^2^. There was some nonlinearity as the maximum depth increased and almost reached breakthrough at a depth value of 1 mm (coating thickness). Breakthrough was achieved at about 4000 impacts per mm^2^ for two out of the three cases.

### 4.3. Calculations of Volume of Emitted Plastic

By considering that all impacts occurred at a 100 m/s speed and using a droplet diameter of 2.4 mm, we could make a conservative estimate for the amount of eroded volume that was emitted from a single turbine. This is because most turbines currently operate at rated tip speeds of less than 100 m/s, and the majority of the droplets during a rain event are measured to have a radius of less than 2 mm [38,39]. Thus, combined with the worse-than-commercial performance of the considered coating in the simulations, the predicted emitted volume was expected to be larger than the actual value for real turbines.

First, we considered an average annual measured rainfall of $H_{total}$ = 1000 mm and an average ground fall velocity for droplets defined as $v_{drop}$. This velocity increases with the size of the falling droplet and ranges between 2 m/s (smaller than 1 mm droplet diameter) and 9 m/s for large droplets [38,39]. In the following, we perform an example calculation of the emitted plastic by considering $v_{drop}$ = 6 m/s. The total rain impingement on the blade, for a constant tangential speed of $V_{blade}$ =100 m/s, would be

(6) $$H_{year}=\frac{V_{blade}}{v_{drop}}H_{total}=\frac{100\;m/s}{6\;m/s}1000\;\mathrm{mm}=16667\;\mathrm{mm}$$

By considering that a coating depth of 1 mm was reached after 4000 impacts/mm^2^ and converting this value to an equivalent impingement over an area of 1 mm^2^, we obtained

(7) $$H_{total}=\left(4000\;{\mathrm{mm}}^{-2}\right)\frac{\pi d^{3}}{6}=28953\;\mathrm{mm}$$

where *d* = 2.4 mm for the droplet diameter. We then converted this value to the equivalent time in years:

(8) $$N_{years}=\frac{H_{total}}{H_{year}}=1.74\;\mathrm{years}$$

Thus, the depth after 1.74 years was 1 mm. We then considered a damaged area of 10 m by 5 cm on the blade, as in [2]. We also considered that some parts of the coating over the surface remained undamaged, and we applied a factor of 0.5, considering that 50% of the damaged area experienced erosion damage. By multiplying the damaged area by the erosion depth of 1 mm and the factor of 0.5, we had an eroded volume per blade after 1.74 years equal to 25$\cdot 10^{-5}$ m^3^. When multiplying by the coating density of 1100 kg/m^3^ and dividing by 1.74 years, we had a mass loss of 159 g per year per blade, or 0.477 kg per turbine per year. This value is similar to those reported in [1,2]. The progression of erosion depth was approximated to be linear from 0 years to 1.74 years, and it was assumed that repairs took place once breakthrough was reached so the same pattern applied throughout the total expected service life of the turbine.

**Figure 15:**
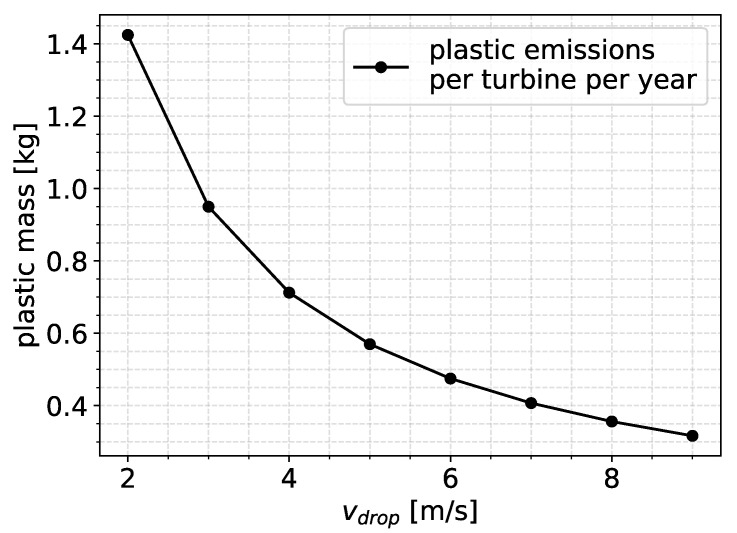
Predicted average microplastic emissions per turbine per year as a function of $v_{drop}$.

However, the value of $v_{drop}$= 6 m/s corresponded to medium-sized droplets, and smaller droplet sizes to the order of 1 mm were much more common during rain events. To account for this, we performed the calculations of Equations (6) and (8) and estimated the average annually emitted plastic volume per turbine for $v_{drop}$, ranging between 2 m/s and 9 m/s. The results are presented in Figure 15. The average annual emission of microplastics for the values of $v_{drop}$ that corresponded to smaller size droplets (1 mm) were to the order of 1 kg per year per turbine.

### 4.4. Effect of a Void on the Erosion Process

**Figure 16:**
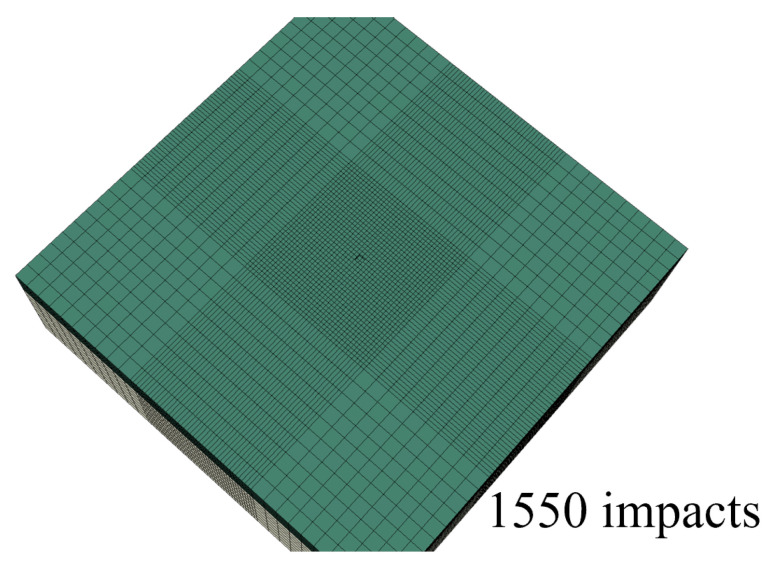

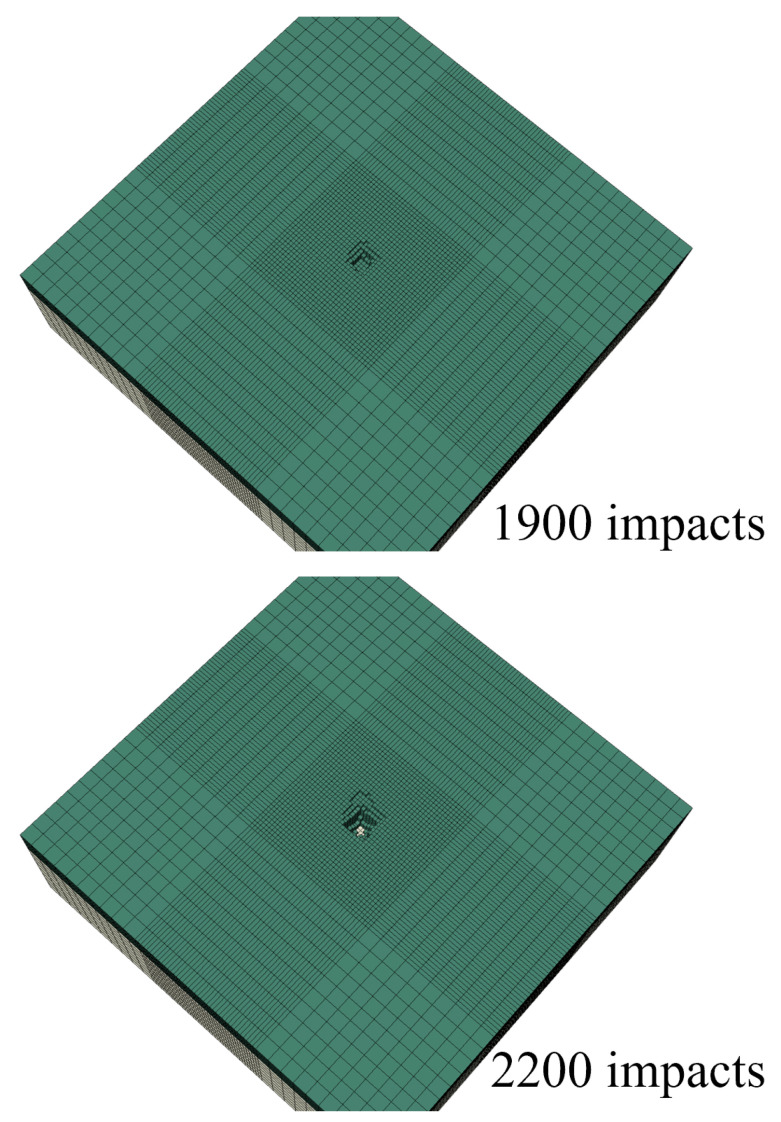
Evolution of surface erosion in the simulations for the case where a 0.2 mm-sized void is present within the coating layer.

**Figure 17:**
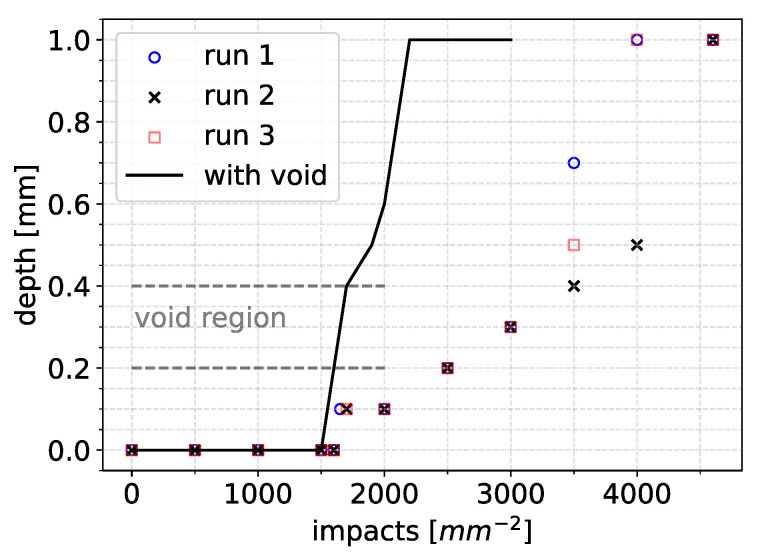
Comparison of the maximum erosion depth evolution curves between the simulation that included a void and those without voids.

In Figure 16, the simulated progression of surface erosion for the model that included a void is presented. The comparison of the erosion depth curves between the cases with and without voids in the coating is presented in Figure 17.

Exposure of the substrate material in the case with the void occurred much quicker than when the coating contained no voids at about 2200 impacts. This was a decrease to the order of 50% when compared with the results of Section 4.2. The end of incubation occurred slightly earlier than when no void was included, but there was a profound difference for the time to breakthrough, as seen in Figure 17. Therefore, voids may significantly reductions in the time to reach breakthrough and trigger different erosion mechanisms, such as interfacial delamination, much earlier.

## 5. Discussion

The modeling framework presented here used only a single impact speed and droplet size value for all impacts. In this study, a constant droplet impact speed of 100 m/s, a normal impact angle for all droplets and a constant droplet diameter of 2.4 mm were used when calculating the microplastic emissions. These conditions were based on a constant tip speed of 100 m/s for a large wind turbine blade, which is a worst-case scenario as the wind turbine does not always operate at the rated tip speed. We also assumed a worst-case scenario for the droplet sizes where all droplets had a large diameter of 2.4 mm, whereas in realistic rain events, the majority of the droplets are less than 2 mm in size. Additionally, the normal impact angle to the surface is widely considered to result in higher mass loss rates. These values offer a worst-case estimate and, at the same time, reduce the variability in the parameters given as input to the FE simulation. Of course, using the developed FE model, one can study the histories of various droplet sizes, impact angles and impact speeds and conduct several what-if analyses.This can be easily expanded by including sampling of those values based on the measured distributions in the Abaqus 2023 Python scripting environment code that generates the FE model for each impact. The same is true for various impact angles. However, this deviated from the scope of this manuscript and could be studied in future work.

The presented approach could also benefit from a more rigorous and automatic way of calculating or fitting the damage equation parameters based on rain erosion testing data, such as that presented in Figure 12. Our current computational resources prohibited us from using a mesh size that would lead to converged results for the stress field in the coating. With more powerful computers and the latest advances in computing power, the presented approach could combine both fine mesh sizes and fast simulation times. The choice of mesh size in this paper was a compromise between solution accuracy and computational time. Fine mesh size simulations require hours to complete with research-level computers. Using those simulations would make it impossible to simulate thousands of droplet impacts. Since the computed stresses in the target material have not reached mesh-independent values, the damage accumulation parameters were expected to change both with the change in the target mesh size and also the droplet mesh size (or the number of SPH particles in our case). Therefore, it is recommended to choose an appropriate mesh size that leads to acceptable solution accuracy with the available resources and calibrate the damage accumulation parameters for that specific mesh size.

This approach models failure of the coating using continuum damage mechanics and element deletion. This is effective when the size of cracks and damage is small and material failure spans over the length of a few elements. However, in some cases, large cracks that cover lengths comparable to the thickness of the coating layer can be observed experimentally. In such cases, the cracks can greatly influence the stress field during droplet impacts, and the continuum approach may no longer be valid. An approach to explicitly include the initiation and growth of “macroscopic” cracks would be required in those cases. The approach presented in this paper is more suitable for cases where erosion is driven by wear of the material and more uniform erosion patterns are expected. In cases where material failure is driven by crack growth initiating from geometric flaws, while some useful results can still be obtained, the approach needs further enhancements which specifically aim for crack growth modeling. Examples of such approaches include the implementation of non-local averaging schemes within the continuum damage mechanics framework for damage initiation and failure. Such approaches could allow the model to simulate the initiation and growth of large cracks compared to the thickness of the coating.

Cycle jumping techniques [31] could be quite useful for reducing the total simulation time and reducing the number of impacts that need to be simulated, as demonstrated in Section 3. However, since droplet impacts do not always occur in the same place, and their touchdown positions are random in a realistic rain event, cycle jumping could introduce spurious damage accumulation. This can be even more pronounced during the mass loss stage where droplet jetting can lead to increased stress values. Perhaps a small value for cycle jumps to the order of 2–3 could provide accurate results and reduce the total simulation time significantly. To examine whether one could use cycle jumping for random impact positions, a numerical study can be conducted where one uses the random impacts FE model to calculate damage accumulation for all impacts and then apply different cycle jumping values to observe whether the results converge for a specific $N_{jump}$ value.

## 6. Conclusions

In this paper, we developed a multiple water droplet impact finite element framework that was used to study rain erosion of wind turbine blade coatings. The framework can model both the erosion incubation period and the mass loss period, and it can account for the interaction between droplet jetting and the eroded coating geometry during impacts. The model was validated with literature data for water droplet erosion of PMMA. We applied the framework to estimate the emissions of microplastics from turbines due to blade erosion for adverse weather and operational conditions, and our worst-case estimate was to the order of 340 g per blade per year. This value is in agreement with the values reported in previous studies. The developed framework is also capable of modeling the effect of flaws in the coating on the erosion process. To demonstrate this, we included a void material region below the coating surface, and this led to a faster progression of erosion in that region. In general, this framework can be used for studying the effects of various types of flaws during both the incubation and mass loss stages of erosion.

## Figures and Tables

**Table 1:** Parameters of the Prony series terms for the viscoelastic coating material model.

$\tau_{i}$ [*s*]	$g_{i}$ [-]
1.00 × 10^9^	6.73 × 10^−5^
1.00 × 10^8^	0.000116
1.00 × 10^7^	0.00059
1.00 × 10^6^	0.001329
100,000	0.005578
10,000	0.025582
1000	0.059994
100	0.122654
10	0.134387
1	0.164244
0.1	0.112804
0.01	0.104109
0.001	0.083982
0.0001	0.060542
1.00 × 10^−5^	0.050356
1.00 × 10^−6^	0.072696

**Table 2:** Calculated number of impacts to the end of incubation form the random impact simulation framework for 100 m/s and 120 m/s impact speeds, with three runs for each impact speed.

Impact Speed	Incubation-Run 1	Incubation-Run 2	Incubation-Run 3	Avg.
120 m/s	450	400	400	417 ± 29
100 m/s	1650	1700	1700	1683 ± 29

## Data Availability

The original contributions presented in this study are included in the article. Further inquiries can be directed to the corresponding authors.
